# Functional magnetic resonance spectroscopy of prolonged motor activation using conventional and spectral GLM analyses

**DOI:** 10.1162/imag_a_00452

**Published:** 2025-01-24

**Authors:** Maria Morelli, Katarzyna Dudzikowska, Dinesh K. Deelchand, Andrew J. Quinn, Paul G. Mullins, Matthew A. J. Apps, Martin Wilson

**Affiliations:** Centre for Human Brain Health and School of Psychology, University of Birmingham, Birmingham, United Kingdom; Center for Magnetic Resonance Research and Department of Radiology, University of Minnesota Medical School, Minneapolis, MN, United States; School of Psychology, Bangor University, Gwynedd, United Kingdom

**Keywords:** fMRS, semi-LASER, lactate, glutamate, aspartate, ABfit

## Abstract

Functional MRS (fMRS) is a technique used to measure metabolic changes in response to increased neuronal activity, providing unique insights into neurotransmitter dynamics and neuroenergetics. In this study, we investigate the response of lactate and glutamate levels in the motor cortex during a sustained motor task using conventional spectral fitting and explore the use of a novel analysis approach based on the application of linear modelling directly to the spectro-temporal fMRS data. fMRS data were acquired at a field strength of 3 Tesla from 23 healthy participants using a short echo-time (28 ms) semi-LASER sequence. The functional task involved rhythmic hand clenching over a duration of 8 min and standard MRS preprocessing steps, including frequency and phase alignment, were employed. Both conventional spectral fitting and direct linear modelling were applied, and results from participant-averaged spectra and metabolite-averaged individual analyses were compared. We observed a 20% increase in lactate in response to the motor task, from participant-averaged spectral fitting, consistent with findings at higher magnetic field strengths. However, statistical testing showed some variability between the two averaging schemes and fitting algorithms. While lactate changes were supported by the direct spectral modelling approach, smaller increases in glutamate (2%) were inconsistent. Exploratory spectral modelling identified a 4% decrease in aspartate, aligning with conventional fitting and observations from prolonged visual stimulation. We demonstrate that lactate dynamics in response to a prolonged motor task are observed using short-echo time semi-LASER at 3 Tesla, and that direct linear modelling of fMRS data is a useful complement to conventional analysis. Future work includes mitigating spectral confounds, such as scalp lipid contamination and lineshape drift, and further validation of our novel direct linear modelling approach through experimental and simulated datasets.

## 1 Introduction

Functional Magnetic Resonance Spectroscopy (fMRS) is an increasingly popular technique to measure dynamic changes in metabolite levels in response to stimuli (Mullins, 2018;Stanley & Raz, 2018). While the method suffers from poorer sensitivity, and therefore spatial resolution compared to functional MRI (fMRI), it offers a uniquely direct insight to the metabolic processes implicated in neuronal activation. One of the earliest^1^H fMRS studies demonstrated a significant elevation in visual cortex lactate levels following prolonged (12 min and longer) visual stimulation at a field strength of 2.1 Tesla (Prichard et al., 1991). These findings have since been replicated by other groups, using various MRS acquisition techniques and field strengths (Bednařík et al., 2015;Maddock et al., 2006;Mangia, Tkác, Gruetter, et al., 2007;Mangia, Tkác, Logothetis, et al., 2007;Sappey-Marinier et al., 1992;Schaller et al., 2013), with reported participant average increases in lactate levels varying between 19% and 100% of resting levels.

The role of lactate in the brain is directly linked to the metabolism of circulatory glucose to meet neuronal energy demands. While there is general agreement on glucose acting as the primary energy substrate for brain tissue in the resting state, the energetics of elevated neuronal activity are less well understood. Initially regarded as an unwanted byproduct of anaerobic glycolysis, lactate has been more recently proposed as a significant neuronal energy source and signalling molecule associated with plasticity and excitability (Magistretti & Allaman, 2018). While the true importance of lactate to elevated neuronal activity has been controversial (Díaz-García et al., 2017), recent evidence suggests the primary neuronal energy substrate may change from glucose to lactate only during particularly demanding tasks (Dembitskaya et al., 2022), potentially explaining some of the apparently conflicting results.

Increases in MR field strength and improved MRS methodology have enhanced the detection of glutamate and Gamma-Aminobutyric Acid (GABA), the primary excitatory and inhibitory neurotransmitters respectively, presenting compelling targets for fMRS studies. fMRS studies of the primary visual cortex at 7T have shown that prolonged visual stimulation induces increased levels of glutamate and lactate, and reductions in glucose and aspartate (Bednařík et al., 2015;Schaller et al., 2013). Prolonged motor activation has also been studied with fMRS, showing task-induced increases in glutamate and lactate in the primary motor cortex (Schaller et al., 2013;Volovyk & Tal, 2020)—findings consistent with prolonged visual stimulation. The MEGA-PRESS MRS sequence (Mescher et al., 1998) is a popular approach for detecting metabolites with weakly J-coupled spins, and may be optimised to measure GABA or lactate by reducing spectral overlap and artefacts from scalp lipids. This approach has been applied to fMRS of the primary motor cortex, detecting an increase in lactate (Koush et al., 2019) and a decrease in GABA (Chen et al., 2017) in response to prolonged activation.

While high field (7 Tesla and above) MRS offers improved SNR and reduced spectral overlap, 3 Tesla MR systems are currently more widely available, and offer a simpler transition from the research setting to clinical use. Despite the popularity of 3 Tesla for neuroimaging applications, most vendor-supplied single-voxel MRS implementations are currently based on the PRESS sequence (Bottomley, 1987), which is known to suffer from localisation inaccuracies and signal loss—particularly for lactate (Yablonskiy et al., 1998). The clinical MRS research community has established a consensus on the use of the semi-LASER sequence over PRESS at 3 Tesla to resolve these technical limitations (Deelchand et al., 2018;Oz & Tkáč, 2011;Wilson et al., 2019); however, the application of semi-LASER to fMRS remains underexplored, despite its potential advantages.

In this study, we employ short echo-time semi-LASER MRS to measure metabolic changes in the primary motor cortex during a prolonged task at a field strength of 3 Tesla. Compared to conventional PRESS, we expect improved estimates of glutamate, due to reduced evolution of strongly J-coupled spins associated with the train of refocusing pulses used in semi-LASER, and improved lactate estimates due to reduced chemical shift displacement. Both these molecules are known to change in response to brain activation, suggesting short echo-time semi-LASER MRS may be a particularly effective acquisition method for fMRS studies at 3 Tesla. As a secondary goal, we introduce a novel analysis approach based on the application of the General Linear Model (GLM) directly to fMRS spectral time-courses and demonstrate how it may be used to corroborate conventional fMRS analysis. This “mass-univariate” GLM approach is widely used for fMRI analysis, and is gaining popularity for spectral analysis of electrophysiology data (Quinn et al., 2024).

## 2 Methods

### 2.1 Participants and functional task

A total of 23 (17 female) right-handed participants with a mean age of 21.6 years were recruited for the study. The study was conducted according to the principles expressed in the Declaration of Helsinki, and participants gave written informed consent before data collection.

At the start of each session, participants were introduced to the MR environment and instructed on the two tasks to be performed inside the scanner. The primary experiment, to be completed concurrently with fMRS acquisition, was to apply rhythmic clenching to a dynamometer held in the right hand, with the participant’s right arm relaxed and by their side in a supine body position. Participants were instructed to pay attention to a fixation cross for 3 min in the first “rest” phase of the experiment. In the following “task” phase, lasting a total of 8 min, participants followed visual prompts to apply a rhythmic squeezing force to the dynamometer. Squeezing was instructed at a rate of 1 Hz by repeatedly displaying the words “squeeze” and “relax”, with durations of 0.7 and 0.3 s respectively (Chen et al., 2017). Participants were instructed to squeeze at each prompt with approximately half their full strength. Finally, a second “rest” phase, lasting 14 min, was completed. The total fMRS experiment lasted 25 min.

A secondary experiment was completed concurrently with fMRI acquisition and constituted a 30-s “rest” block, followed by a 30-s “task” block and a final 60-s “rest” block. These data were acquired to ensure the fMRS voxel contained brain tissue hemodynamically responsive to the hand-clenching task. All other aspects of the secondary task were identical to the first.

The MR-compatible dynamometer was combined with a compatible amplifier interface, laptop, and associated software (BIOPAC Systems, Inc, CA, USA) for real-time monitoring of the applied force to confirm participants were performing the two experiments correctly.

Visual stimuli were presented using Psychopy software (Peirce et al., 2019) version 2023.1 onto a projector screen positioned inside the scanner bore and behind the participant’s head—viewed via a mirror attached to the head-coil. A Propixx system (VPixx Technologies Inc, Canada) was situated outside the scanner room and used to project images onto the screen via a waveguide in line with the scanner bore.

### 2.2 MR acquisition

MR data were acquired with a 3 Tesla Siemens Magnetom Prisma (Siemens Healthcare, Erlangen, Germany) system using a 32-channel receiver head coil array. A T1-weighted MRI scan was acquired sagittally with a 3D-MPRAGE sequence: FOV = 208 × 256 × 256 mm, resolution = 1 × 1 × 1 mm, TE / TR = 2 ms / 2000 ms, inversion time = 880 ms, flip angle = 8°, and GRAPPA acceleration factor = 2 (4 min 54 s scan duration).

**Fig. 1.**
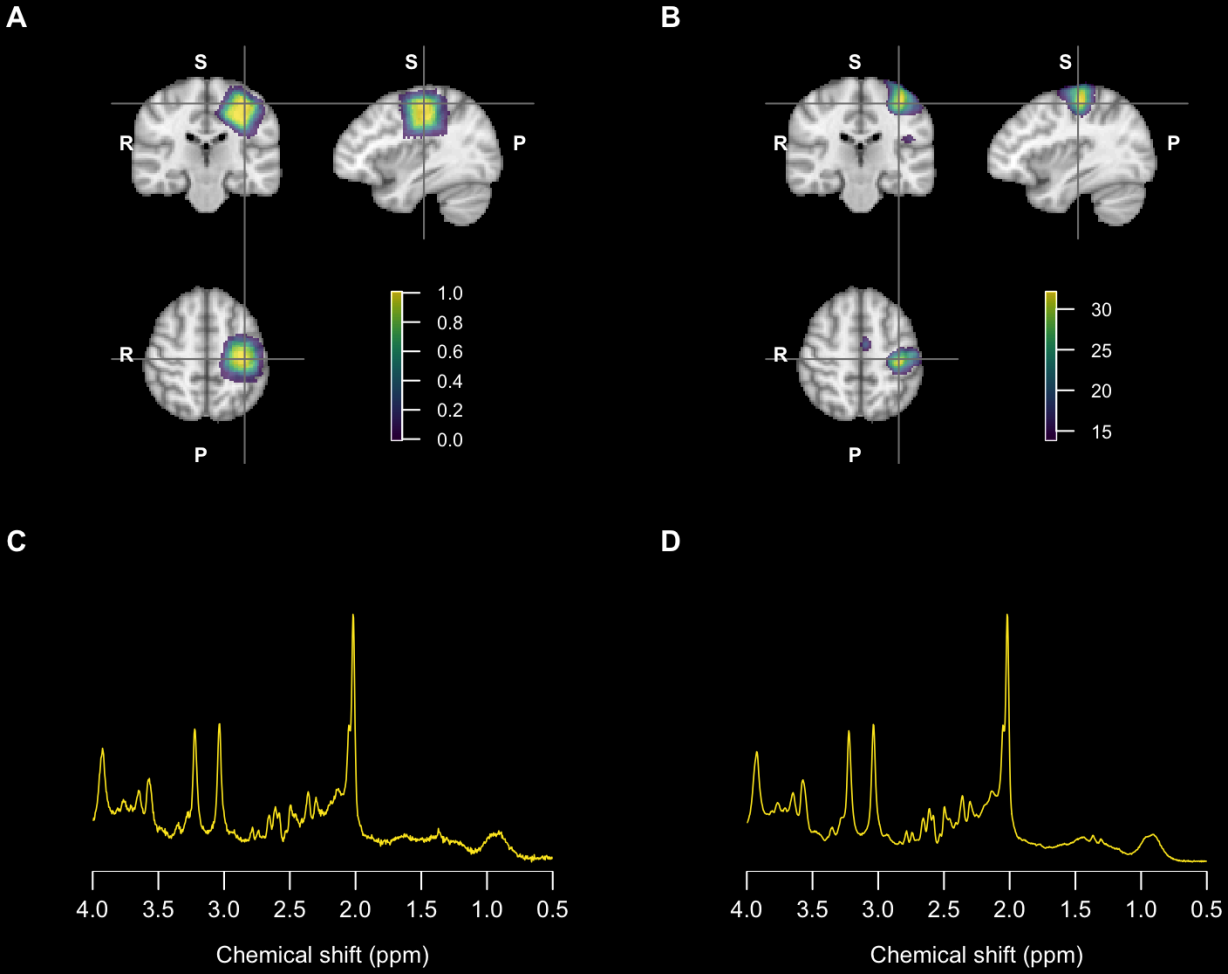
(A) Mean fMRS voxel location overlaid on the MNI152 template. (B) Group-level fMRI BOLD activation map (Z-statistic) overlaid on the MNI152 template with identical slice positions to part (A). (C) mean spectrum averaged over 50 transients following frequency and phase correction. (D) mean spectrum averaged over all transients (N = 750) following frequency and phase correction.

Single-voxel fMRS was performed using the semi-LASER sequence (Deelchand et al., 2018) with a 25 mm-sided cubic excitation region. Seven hundred and fifty transients were acquired with a 90-degree flip angle and an 8-step phase cycling scheme; TE / TR = 28 ms / 2000 ms and VAPOR water suppression (25 min scan duration). Four transients were also acquired without VAPOR water suppression. B_0_shimming was performed using the “brain” method implemented by the vendor. MRS data were exported and converted to NIfTI MRS format (Clarke et al., 2022) as 2048 complex data points acquired with a spectral width of 2000 Hz. The MRS voxel was positioned over the hand motor cortex region in the left hemisphere based on established anatomical landmarks (Caulo et al., 2007) identified from the immediately preceding T1-weighted images (Fig. 1A).

fMRI data were acquired using a multi-band accelerated GRE-EPI sequence with an isotropic voxel resolution of 2.5 mm over a 210 x 210 x 142.5 mm FOV (AP x LR x FH). Eighty fMRI volumes (each containing 57 transverse slices) were acquired with a TR of 1500 ms; TE of 35 ms; 71 degree excitation flip angle; A>>P phase encoding direction; and multi-band acceleration factor of 3 (2 min scan duration).

Trigger pulses were generated by the fMRS and fMRI sequences at each TR to ensure temporal synchronisation between the functional tasks and data acquisition. Padding was applied to the inside of the head coil to aid participant comfort and minimise head motion.

### 2.3 Conventional fMRS analysis

Temporal instability in the frequency offset and zero-order phase for each dynamic spectrum was corrected using the RATS method (Wilson, 2019). The 4–1.9 ppm range (containing the strongest metabolite signals) from the mean uncorrected spectrum calculated over the 750 transients for each participant was used as the reference spectrum for correction. The mean corrected spectrum for each participant was then further frequency- and phase-aligned to a simple simulated spectrum composed of three resonances with equal intensity at 2.01, 3.03, and 3.22 ppm with 4 Hz Lorentzian line-broadening applied—using the RATS method. These “participant-level” frequency and phase corrections were then applied back to the individually corrected dynamics to ensure that every single spectrum was consistently phase- and frequency-aligned across all participants. Dynamic spectra from each participant were amplitude normalised to the peak height of the tCr resonance at 3.03 ppm measured from the dynamic mean spectrum on a per-participant basis.

fMRS data quality was assessed by: 1) visual inspection of mean spectra; 2) dynamic spectrograms; 3) spectral SNR based on the peak height of the tNAA resonance; and 4) linewidth of the tNAA resonance at 2.01 ppm.

For spectral fitting, corrected spectra were averaged into temporal blocks of 50 spectra to enhance spectral SNR, resulting in 15 dynamic spectra per participant. Conventional spectral fitting was performed for each participant separately, and a further analysis was performed following averaging the 15 dynamic spectra across all participants.

Spectral fitting was performed using a simulated basis containing the following set of standard brain metabolites: alanine (Ala), aspartate (Asp), creatine (Cr), gamma-Aminobutyric acid (GABA), glucose (Glc), glutamine (Gln), glutathione (GSH), glutamine (Gln), glycine (Gly), glycerophosphocholine (GPC), myo-inositol (Ins), lactate (Lac), N-acetylaspartate (NAA), N-acetylaspartylglutamate (NAAG), phosphocholine (PCho), phosphocreatine (PCr), phophosphoethanolamine (PEth), scyllo-inositol (sIns), and taurine (Tau). The commonly used set of simulated broad signals to model macromolecular and lipid signals present in^1^H MRS data were also included in the basis; see Table 1 ofWilson et al. (2011)for the full listing.

Spectral fitting was performed initially using the ABfit algorithm (Wilson, 2021a) and repeated using LCModel (Provencher, 1993) to explore consistency of findings across two different methods. Both algorithms were applied with default fitting parameters and an identical basis set as described above. Metabolite levels were divided by the tCr level (sum of PCr and Cr) estimated from the mean spectrum across all time points.

### 2.4 Exploratory fMRS spectro-temporal statistical modelling

The standard approach for fMRS analysis involves temporal averaging of spectra to boost SNR, and therefore the accuracy of metabolite estimates, followed by the use of conventional spectral fitting algorithms to measure dynamic metabolite changes. More recently, we have shown how the application of multiple univariate statistical tests directly to individual MRS data points can support findings from spectral fitting, and potentially reveal novel information on individual differences in neurometabolic profiles (Vella et al., 2023;Wu et al., 2022). A similar approach may be easily adapted to fMRS, where each frequency domain data point can be treated as an independent time course to be fitted with a linear model incorporating the predicted metabolite dynamics. Statistical measures may then be derived from each fit to indicate the likelihood of a given frequency region being associated with a particular functional task. Such an approach is analogous to conventional fMRI analysis—where linear fits incorporating the predicted BOLD response are applied to the time course of each spatially encoded voxel.

Following frequency and phase alignment and normalisation steps described earlier, an additional 2 Hz Gaussian line broadening was applied to enhance spectral SNR. Spectra were cropped to the region between 0 and 4.3 ppm, and asymmetric least squares baseline correction was applied (Eilers & Boelens, 2005). Since the true dynamic responses of lactate and glutamate to visual stimuli are yet to be established, a simple boxcar function was assumed, with values of 0 during rest blocks and 1 in the task block. Linear regression of the boxcar function was applied to each spectral time course for participant-averaged data following the preprocessing steps described insection 2.4. Temporal averaging was not performed.

All spectral processing, fitting, and associated statistics and plots were performed using the spant MRS analysis package (Wilson, 2021b) implemented in the R statistical programming language (R Core Team, 2021). All reported t-test statistics were calculated using the Student’s t-test.

### 2.5 fMRI analysis

BOLD activation maps were generated using FEAT, distributed as part of the FSL software package (Woolrich et al., 2009), version 6.0.6.1. A standard analysis pipeline was used, incorporating motion correction and spatial registration to a defaced T1-weighted MPRAGE anatomical scan. 5 mm of spatial smoothing was applied to the EPI data, and a z-threshold of 5.3 was used to generate statistical maps to confirm concordance between MRS voxel placement and BOLD activation. A group-level fixed-effects analysis was also performed to determine the spatial location of peak BOLD activation (Fig. 1B).

## 3 Results

### 3.1 fMRS data quality

Manual inspection of spectrograms prior to frequency and phase correction revealed significant movement artefacts for 3 of the 23 participants, and these scans were removed from subsequent analysis. An additional scan was removed due to an unusually high degradation in tNAA lineshape FWHM from approximately 0.05 to 0.07 ppm, potentially arising from scanner instability, or gradual participant movement throughout the scan. One participant was unable to complete the fMRI task due to fatigue; however, the fMRS data were still included in subsequent analyses since they were able to complete the associated task, as confirmed by real-time observation of the force applied to the dynamometer. All other participants correctly executed the task during the fMRS and fMRI scans.

The single-shot SNR of each dynamic spectrum was measured and a dynamic median value calculated for each fMRS scan based on the maximum peak height of the tNAA resonance at 2.01 ppm. The mean of these single-shot SNR values was calculated across the 19 good-quality scans as 31 with a standard deviation of 3.4. Similarly, line widths were estimated from the tNAA resonance of the dynamic mean spectrum. The mean linewidth calculated across the 19 good-quality scans was 0.047 ppm with a standard deviation of 0.010 ppm.

Figure 1confirms good spatial concurrence between the mean fMRS voxel location (Fig. 1A) and peak fMRI BOLD activation (Fig. 1B) estimated from a group-level analysis.

### 3.2 Conventional fMRS analysis

Time-courses for participant-averaged spectra are plotted for glutamate and lactate inFigure 2, with a corresponding simplified version shown inFigure S2. Spectral fits for the first time point (average of first 50 transients) of the participant-averaged spectra are shown for ABfit and LCModel inFigure S1. Glutamate levels appear consistent throughout the functional task, whereas lactate shows a steady increase. The mean error (across the 15 dynamics) for glutamate and lactate was estimated as 3.6% and 11.1% respectively, relative to their level at the first time point, using the CRLB method. The corresponding mean fit quality number (Kreis et al., 2020) was 3.7. A t-test between the metabolite levels during the task vs. rest blocks (data points within vs. outside the red boundary inFig. 2) supports this observation, with lactate demonstrating a statistically significant change: t(13) = 5.15, p = 0.00019, in contrast to glutamate: t(13) = 0.24, p = 0.81.

**Fig. 2.**
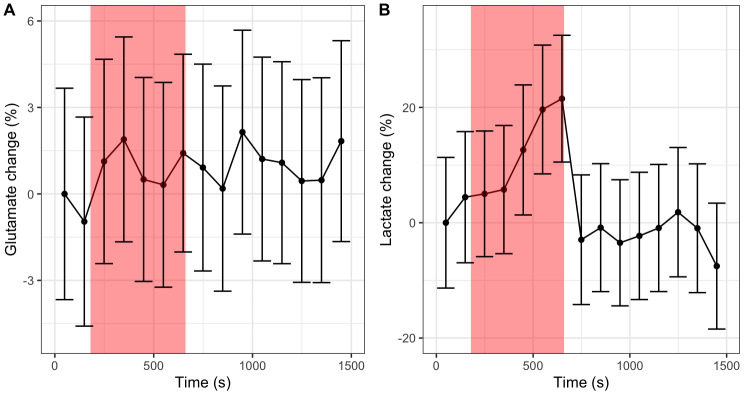
Time-courses for (A) glutamate and (B) lactate estimated from spectral fitting of participant-averaged spectra using the ABfit method. Error bars represent the standard deviation estimated from Cramer-Rao Lower Bounds. The translucent red region represents the task block.

**Fig. 3.**
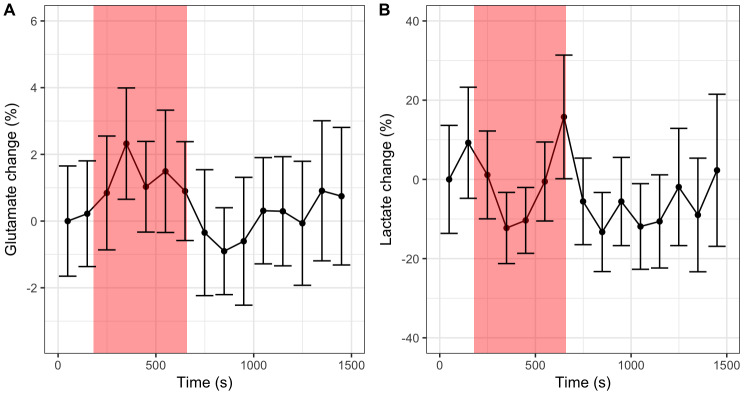
Mean time-courses for (A) glutamate and (B) lactate estimated from spectral fitting of individual participant spectra using the ABfit method. Error bars represent the standard error across participants. The translucent red region represents the task block.

Figure 3shows the mean time-courses for glutamate and lactate calculated over the fitting results derived from each participant separately (simplified version shown inFig. S3). Using this alternate analysis approach reveals a change in statistical significance for glutamate (t(13) = 3.95, p = 0.0017) and lactate (t(13) = 0.72, p = 0.48), between rest and task states, when compared to fitting results from the participant-averaged spectra (Fig. 2). Individual participant time courses are plotted inFigure S4.

**Fig. 4.**
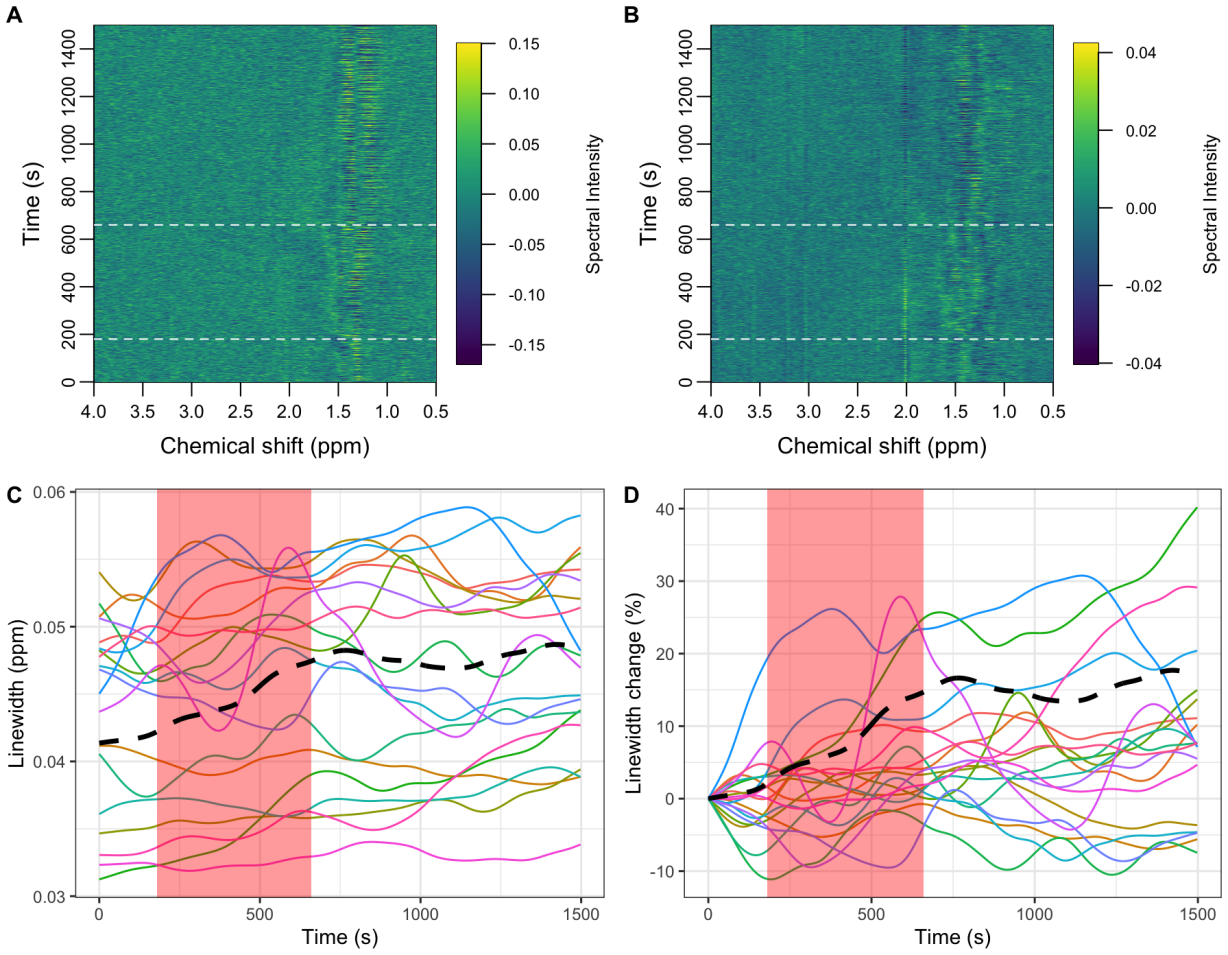
Spectrograms of (A) an example participant fMRS scan, (B) participant-averaged fMRS scan. The dynamic mean spectrum was subtracted from both spectrograms to highlight small temporal variations. Dashed horizontal white lines represent the transitions between rest and task states. (C) the smoothed tNAA linewidth for individual participants (coloured lines) and participant-averaged scan (black dashed). (D) same as (C) but measured as a percentage change from the first time-point.

The discordance between metabolite dynamics estimated from participant mean spectra compared to mean levels from individual participant scans is unexpected, and suggests a level of instability present in the spectral data, the analysis methodology, or both. Spectrograms were generated for each individual participant scan and the mean participant scan to explore sources of temporal variance. The dynamic mean spectrum was subtracted from each spectrogram. 4 Hz Gaussian line-broadening and a linear baseline correction was also applied to each spectrum to aid the visualisation of small temporal changes.Figure 4Ashows a typical example of an individual participant spectrogram, where the primary source of temporal variance is evident in the spectral region between 1 and 1.5 ppm. The smooth spectral appearance, combined with the frequency range, strongly suggests this source of variance originates from out-of-volume scalp lipids—a commonly observed artefact and confound for lactate measurement in^1^H MRS brain data.Figure 4Bshows how this artefact is reduced from approximately ±15% to ±4% the height of the tCr resonance in the participant mean spectrum compared to the individual participant spectrogram inFigure 4A. This potentially explains the disagreement between lactate dynamics observed inFigure 2Bcompared toFigure 3Bas the effectively random phase of the lipid artefact results in its suppression in the participant-averaged spectra.Figure S5shows the mean spectrum for each participant included in the spectral analysis. While some participants had higher levels of visual lipid interference (e.g., sub-01), visual inspection of these spectrograms did not show significantly increased variance in this region and therefore these participants were not excluded from spectral analysis.

In addition to a reduced lipid artefact,Figure 4Bshows temporal changes in the spectral intensity of the primary singlet resonances of tNAA, tCr, and tCho at 2.01, 3.03, and 3.22 ppm respectively. While similar changes in the intensity of these resonances are present in the individual participant data, the improved SNR of the average participant spectra aids visualisation of this type of variability. These changes are attributed to dynamic linewidth variability, potentially resulting from a slow degradation in magnetic field homogeneity, withFigure 4Dshowing gradual lineshape increases of up to 40% in the worst case. The participant average data (dashed black line in parts C and D), however, shows a milder increase of up to 20% (from 0.041 to 0.049 ppm). Spectral fitting approaches have been shown previously to be sensitive to changes in linewidth (Mangia et al., 2006;Near et al., 2013), therefore we propose that differences in SNR and linewidth variability between the individual subject analysis (Fig. 3A) and subject-averaged analysis (Fig. 2A) may explain the differences in estimated glutamate dynamics.

Exploratory analysis was also performed with the LCModel fitting algorithm (Provencher, 1993), since this fitting method is more commonly used for fMRS analysis. Time-courses for glutamate and lactate, measured from the participant mean spectra, are shown inFigure S6. Applying a t-test between the metabolite levels during the task vs. rest blocks gave statistically significant differences for both glutamate (t(13) = 2.41, p = 0.031) and lactate (t(13) = 4.98, p = 0.0003). Consistent results were also found for the analysis of mean time courses from individual participant scans, with statistically significant increases in glutamate (t(13) = 2.67, p = 0.019) and lactate (t(13) = 4.21, p = 0.001) during the task block (Fig. S7).

Minimum reporting standards information for MRS (Lin et al., 2021) is provided inTable S1.

### 3.3 Exploratory fMRS spectro-temporal statistical modelling

**Fig. 5.**
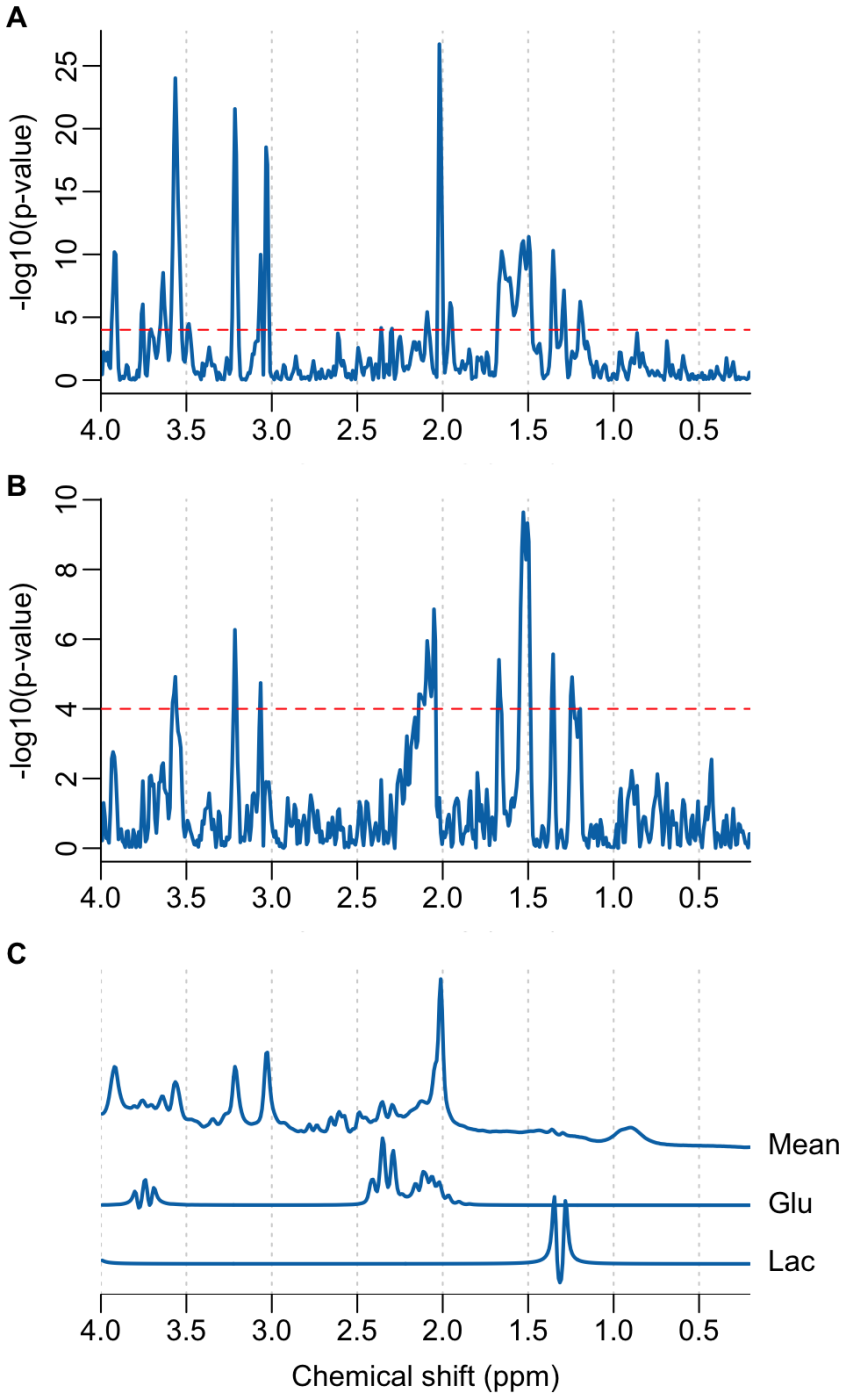
(A) A Manhattan plot illustrating spectral regions temporally associated with the functional task, assuming a simple boxcar function. The dashed horizontal red line represents the Bonferroni-corrected p-value threshold (p < 1e-4). (B) Same as (A) but with an added nuisance regressor to suppress the influence of lineshape variability. (C) Mean fMRS spectrum with simulated glutamate (Glu) and lactate (Lac) signals. Simulated signals are scaled to have similar maximum intensities to aid the assignment of parts (A) and (B).

Further exploratory analysis of the participant mean spectral data was performed using a novel processing approach performed directly on the spectral data points, obviating the need for a simulated basis set or complex fitting algorithm.Figure 5Ahighlights the spectral regions most different between the task and rest states by fitting each spectral time-course with a simple boxcar function corresponding to the task state. In this preliminary analysis, the spectral regions most strongly associated with the task are the three primary singlet resonances of tNAA, tCr, and tCho and the strong myo-inositol multiplet at 3.55 ppm. These differences are due to the drift in linewidth (seeFig. 4) rather than genuine metabolic changes, therefore we added a nuisance regressor to the boxcar function which was calculated from the temporally smoothed time-course of the integrated spectral region between 1.97 to 2.04 ppm.Figure 5Bshows how the incorporation of the nuisance regressor significantly reduces the intensity of the spectral regions most associated with lineshape drift. The primary resonances of lactate at 1.28 and 1.35 ppm are present in both the basic analysis (Fig. 5A) and the analysis including the nuisance component (Fig. 5B), suggesting the lactate changes are not strongly influenced by lineshape changes. Conversely, the primary glutamate resonance at 2.35 ppm shows only a weak statistical association to the task compared to lactate in both analyses.

**Fig. 6.**
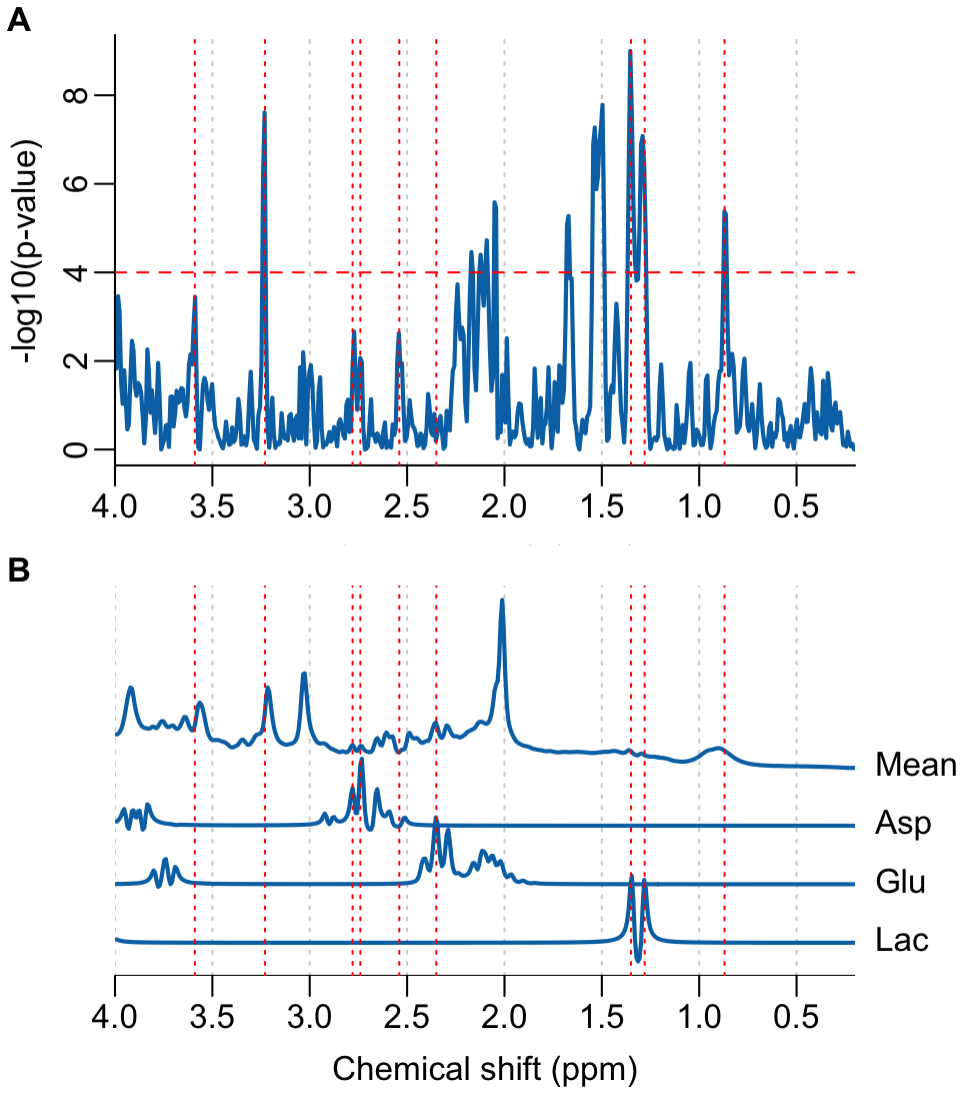
(A) A Manhattan plot illustrating spectral regions temporally associated with the functional task, assuming a simple boxcar function delayed by 120 s. The dashed horizontal red line represents the Bonferroni-corrected p-value threshold (p < 1e-4). (B) mean fMRS spectrum with simulated glutamate (Glu), lactate (Lac), and aspartate (Asp) signals. Simulated signals are scaled to have similar maximum intensities to aid the assignment of part (A). Dashed red lines highlight spectral features at 0.87, 1.28, 1.35, 2.35, 2.54, 2.74, 2.78, 3.23, and 3.59 ppm.

Metabolite dynamics in longer block designs have been shown to have a lagged response to tasks (Bednařík et al., 2015), with some metabolites taking up to 2 min to plateau followed by a slow decline once the task has stopped.Figure 6shows how delaying the boxcar function by 2 min further suppresses the primary singlet spectral regions most sensitive to lineshape changes, while maintaining a strong association with lactate levels. Very little association with the primary resonance of glutamate is apparent with the lagged response function, however we do note a weak association with potential resonances at 2.74 and 2.78 ppm which we tentatively assign to aspartate. A -5.4% decrease in aspartate in response to visual stimulation has been reported previously (Bednařík et al., 2015), therefore we chose to examine these levels estimated from conventional spectral fitting.Figure S8shows the aspartate levels estimated from the participant-averaged spectra. An approximately 4% decrease is seen at the 5th time point in agreement with (Bednařík et al., 2015). Statistical testing between the rest and task time points for aspartate did not reach statistical significance (t(13) = -1.97, p = 0.07); however, delaying the task window by one time point (100 s), similar toFigure 6, did reveal a statistically significant difference (t(13) = -4.59, p = 0.0005). The delayed boxcar regressor weighings (beta weights) are shown inFigure S9Aand confirm a task-related increase in lactate (positive beta weightings at 1.28 and 1.35 ppm) and decrease in aspartate (negative beta weightings at 2.74 and 2.78 ppm).

Figure S10 depicts each regressor used in the spectral GLM analyses.

## 4 Discussion

Previous studies of prolonged motor activation have reported increases in glutamate and lactate in the motor cortex, and our findings largely support these observations (Chen et al., 2017;Koush et al., 2019;Schaller et al., 2013;Volovyk & Tal, 2020). However, we also observed how measures of statistical significance were sensitive to the analysis approach, showing differences depending on how participant-averaged results were compiled and the choice of spectral fitting algorithm. Inspection of spectral variability (Fig. 4) demonstrated that dynamic changes in metabolite linewidth and lipid artefacts were the primary sources of variance and that averaging across participant spectra may reduce these artefacts, potentially explaining some of the inconsistencies between analysis approaches (Fig. 2vs.Fig. 3). Differing results between spectral fitting strategies have been reported previously (Marjańska et al., 2022), and are indicative of the inherent instabilities of non-linear analyses in the presence of noise. While fitting stability may be imposed through various forms of regularisation (e.g., Bayesian priors), this ultimately results in some degree of bias due to the “bias-variance tradeoff”. Even within the same fitting approach, variation in results may arise from minor changes in basis set constituents (Demler et al., 2024), further emphasising the inherent instability of spectral fitting. For large metabolite changes, typically seen with clinical MRS, these instabilities are less problematic; however, changes are typically on the order of less than 10 percent for^1^H fMRS and, therefore, more stable analysis approaches are required.

In this study, we present an alternate analysis approach, based on the GLM, to improve the stability of fMRS findings. In contrast to conventional spectral fitting, no assumptions about the basis-set are made, avoiding one potential source of variability between analyses (Demler et al., 2024). Simple linear modelling is performed directly on spectral data points using the “mass univariate” approach, which is well-established for fMRI, and is becoming a more popular tool for electrophysiology (Quinn et al., 2024) and conventional MRS (Vella et al., 2023;Wu et al., 2022). While further validation of the approach is required, we show here how it may be used to support or question findings from conventional analyses. The corroboration between conventional fitting, which shows an approximately 20% increase in lactate from baseline levels, and the clear doublet appearance at 1.28 and 1.35 ppm in the Manhattan plot (Fig. 6A) adds confidence to these results. A similar increase in lactate in response to a prolonged motor task (17%) was reported at a field strength of 7 Tesla (Schaller et al., 2014) and further supports our findings.

This is the first study to show lactate dynamics of prolonged motor activation may be observed at 3 Tesla. Another similar study at 3 Tesla was unable to measure these changes due to unreliable quantitation (Volovyk & Tal, 2020). Possible explanations for this difference in findings may be due to differences in voxel dimensions (15.6 ml vs. 6 ml) and alternate acquisition methodologies (semi-LASER vs. PR-STRESS). While larger voxel dimensions are expected to be more susceptible to scalp lipid contamination, this may be offset by improved SNR. Our results may also suggest that the spatial extent of the lactate response extends beyond a 6 ml volume, since unaffected tissue would effectively “dilute” the measured percentage change, yet we still observe a relatively large change of 20%—consistent with 7 Tesla studies.

Our new GLM-based approach involves performing hundreds of statistical tests, and, therefore, the risk of detecting false positives (type I error) needs to be considered. For this study we have chosen to display the statistical spectra together with a Bonferroni-corrected threshold of significance to aid the visual assessment of spectral changes. However, Bonferroni correction is known to be overly conservative, particularly in our case where there are inherent correlations between spectral points. In fMRS, these correlations arise from a number of distinct sources: 1) single metabolite resonances span multiple spectral data points due to intrinsic (T_2_relaxation) and extrinsic (B_0_inhomogeneity) factors; 2) metabolites often give rise to multiple resonances, either clustered together (multiplets) or with greater frequency separation; 3) applied linebroadening, to improve spectral SNR, will introduce correlations between nearby spectral data points, even if they originate purely from noise; and 4) artefacts, such as dynamic baseline variability. While some of these correlations have the potential to reduce type I errors, for example by assigning higher significance to “spectral-clusters” corresponding to anticipated metabolite resonance spectral widths, these need to be carefully designed and implemented to avoid spurious results (Eklund et al., 2016).

Amelioration of type I errors is a well-explored area of fMRI analysis research, where multiple comparisons are typically performed across the three spatial dimensions, rather than the single spectral dimension considered here for single-voxel fMRS. Two of the most popular approaches employed in fMRI are 1) random field theory (T. Nichols & Hayasaka, 2003), based on assumptions on the intrinsic spatial smoothness of the data, and 2) permutation testing (T. E. Nichols & Holmes, 2002), based on the assumption that data points are “exchangeable” under the null hypothesis. Both of these methods could be adapted to our proposed spectral GLM method; however, in the absence of the “ground-truth”, we only present the conservative Bonferroni threshold here. Future work will focus on accurate fMRS simulations to validate more sophisticated approaches.

Glutamate changes of approximately 2%, as measured from spectral fitting (Fig. 3A), were comparatively smaller than the values of around 4% reported elsewhere at 3 Tesla (Volovyk & Tal, 2020). We also found inconsistency between the analysis of participant average spectra (Fig. 2A), the participant-averaged metabolite levels (Fig. 3B), and the GLM spectral modelling (Fig. 6A). While multiple resonances around 2.2 ppm inFigure 6Aare consistent with glutamate (Fig. 6B), the strongest resonances expected around 2.35 ppm are absent. This may imply that fitting differences are driven by changes in the unassigned resonances at 2.2 ppm, rather than glutamate, or that lineshape variance is correlated with glutamate changes, obscuring detection accuracy. We also note that our voxel volume of 15.6 ml was larger than in similar studies (~10 ml), which may suggest that glutamate changes have a smaller spatial extent than lactate.

The exploratory spectral GLM analysis (Fig. 6A) highlighted a potential metabolic response in resonances at 2.74 and 2.78 ppm. These frequencies are in good agreement with those expected from aspartate, prompting further exploratory analysis of the conventionally fitted data. An approximately 4% decrease in aspartate is shown inFigure S8, consistent in magnitude and direction to changes observed during extended visual stimulation at 7 Tesla (Bednařík et al., 2015). While exploratory in nature, the agreement between spectral GLM, conventional analysis, and high field studies suggests the change is likely genuine and demonstrates how novel analysis approaches have the potential to improve the sensitivity and reliability of fMRS.

In addition to those already mentioned,Figure 6Ashows a number of other spectral regions with a statistical dependence on the functional task. The peak at 3.23 ppm is assigned to resonances from choline, phosphocholine, and glycerophosphocholine and previous work has shown alterations in choline levels during reversal learning (Bell et al., 2018). While this resonance is also known to be sensitive to lineshape alterations (Fig. 5A), the absence of a similar artefact in the creatine region suggests a change in concentration or frequency shift of the choline resonances may be genuine, and warrants further study. Further resonances at 0.87, 1.50, 1.68, 2.54, and 3.59 ppm cannot be assigned with confidence; however, we note some similarity to resonances expected from branched-chain amino acids.

In the participant-averaged data, we see good agreement for lactate changes between ABfit, LCModel, and our GLM-based approach, with each method showing a statistically significant increase in response to the task. For glutamate, agreement is mixed, with LCModel showing a statistically significant increase in the participant-averaged data, whereas ABfit and the GLM-based approach did not show a convincing change.Figure S1shows example fits for LCModel and ABfit, with both methods showing similar residuals and baselines. Despite visually similar fits, a number of differences exist between the design and default behaviour of the two methods. LCModel has a more flexible lineshape model, with smoothness being the only constraint, whereas ABfit assumes a simpler asymmetric Voigt model. LCModel also incorporates soft constraints between metabolite levels, which in some cases introduces stability from noise and in others may result in biassed estimates. These differences may explain some of the conflicting results presented here, and further work is required to determine which algorithmic aspects are beneficial for typical fMRS applications.

Alternative MRS analysis approaches involve 2D fitting, whereby multiple spectra are fit to a metabolite basis simultaneously—reducing the number of parameters requiring optimisation, resulting in more accurate estimates. While the advantages of 2D fitting in MRS have been long established in various contexts (Chong et al., 2011;Schulte & Boesiger, 2006;Van Ormondt et al., 1990) there has been renewed interest in the application of 2D fitting to functional and diffusion MRS (Clarke et al., 2024;Tal, 2023). Other approaches involve combining conventional 1D fitting with a GLM applied to the metabolite time-courses (Ligneul et al., 2021). How these methods compare to the direct linear modelling of spectral time-courses introduced here, particularly in the presence of confounding temporal instabilities, presents a key area for the future development of fMRS analysis methodology.

## 5 Conclusions

We have shown that lactate dynamics in response to a prolonged motor task are observed using short-echo time semi-LASER at 3 Tesla. A novel approach for fMRS data analysis is introduced, based on direct linear modelling of spectral time-courses, demonstrating potential for measuring small changes associated with aspartate and other unassigned molecules. Future work includes reducing the effect of spectral confounds, such as scalp lipid contamination and temporal lineshape variability, and further validation of our new analysis approach using experimentally acquired and simulated datasets.

## Supplementary Material

Supplementary Material

## Data Availability

Code to generate the results presented in this paper is available from:https://github.com/martin3141/fmrs_motor_paper Data to generate the results presented in this paper are available from:https://doi.org/10.5281/zenodo.11190358
