# Supplementary material for: Functional magnetic resonance spectroscopy of prolonged motor activation using conventional and spectral GLM analyses
Source: Imaging Neurosci (Camb). 2025 Jan 24;3:imag_a_00452. doi: 10.1162/imag_a_00452 (PMC12319857; doi:10.1162/imag_a_00452)
Supplement: Supplementary Material [file imag_a_00452-supp.pdf]

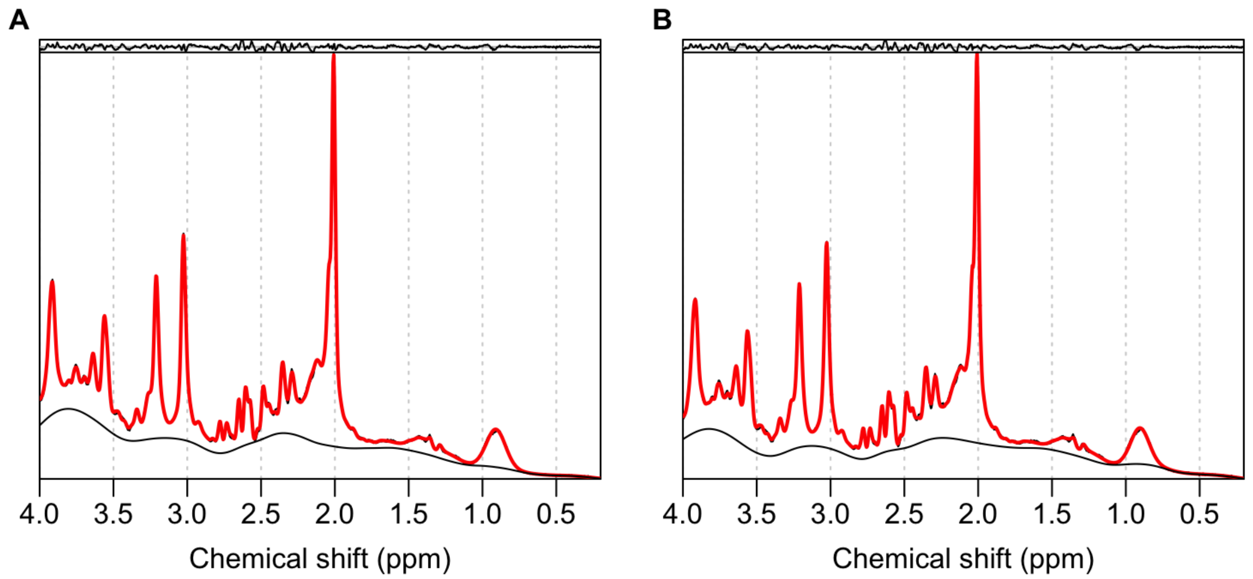

Fig. S1 Spectral fits for the first time point (mean over the first 50 dynamics) of the participant-averaged spectra are shown for A) ABfit and B) LCModel.

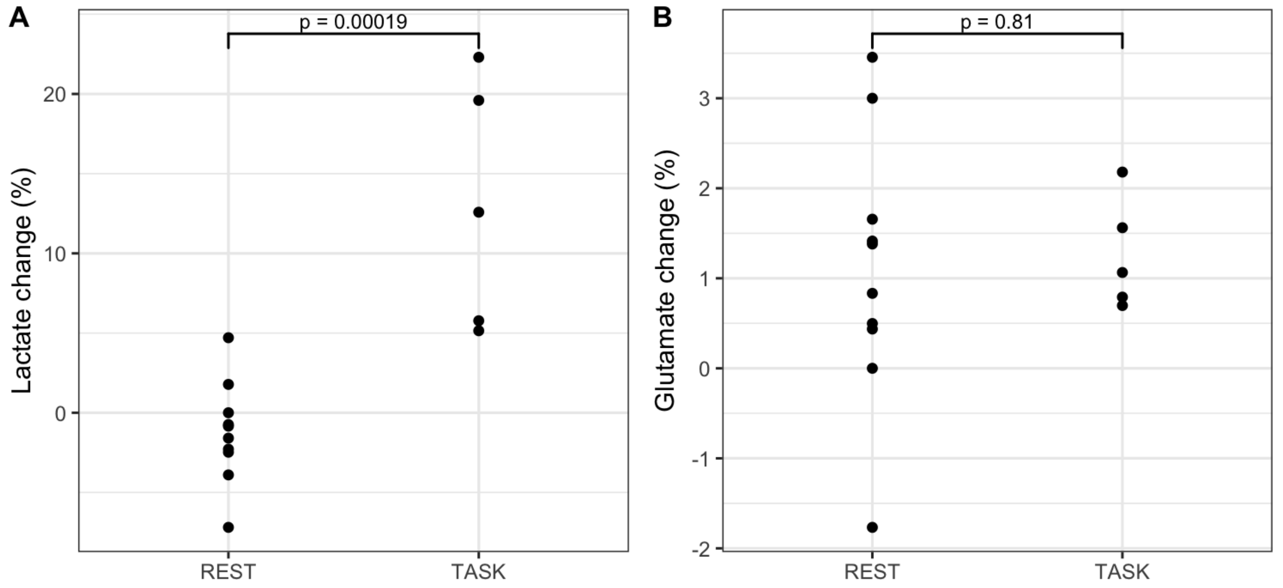

Fig. S2 Simplified version of Fig 2 (omitting temporal information) to aid visual comparison between rest and task states. p values have been calculated using the Student's t-test between the two states.

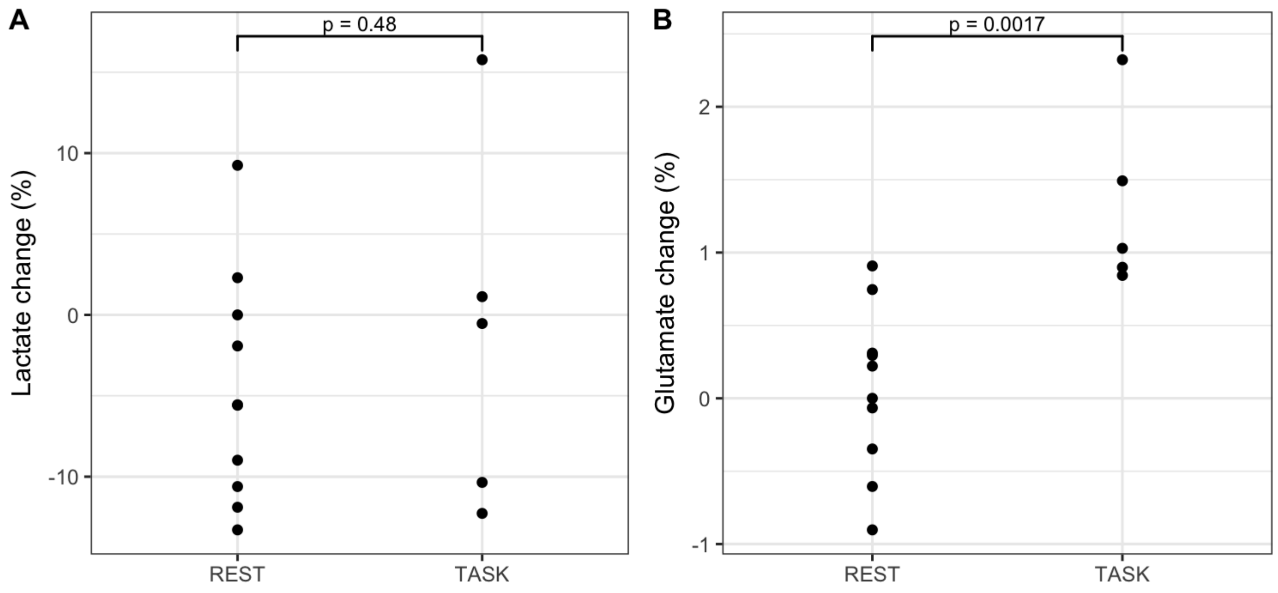

Fig. S3 Simplified version of Fig 3 (omitting temporal information) to aid visual comparison between rest and task states. p values have been calculated using the Student's t-test between the two states.

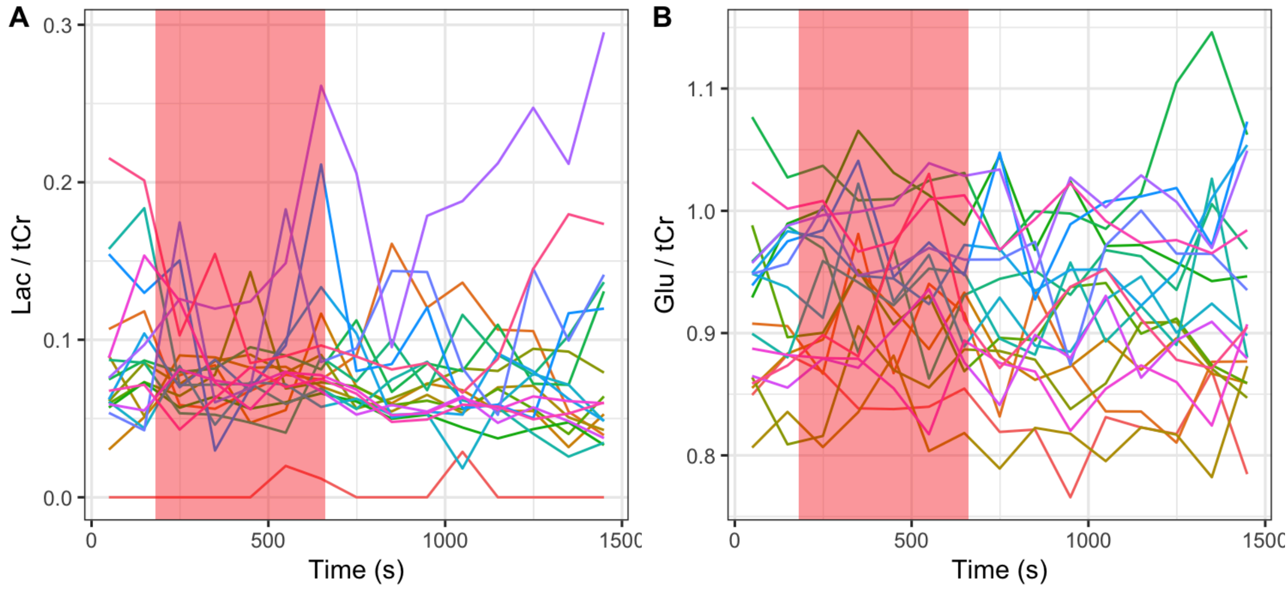

Fig. S4 Individual participant A) lactate and B) glutamate levels estimated with ABfit.

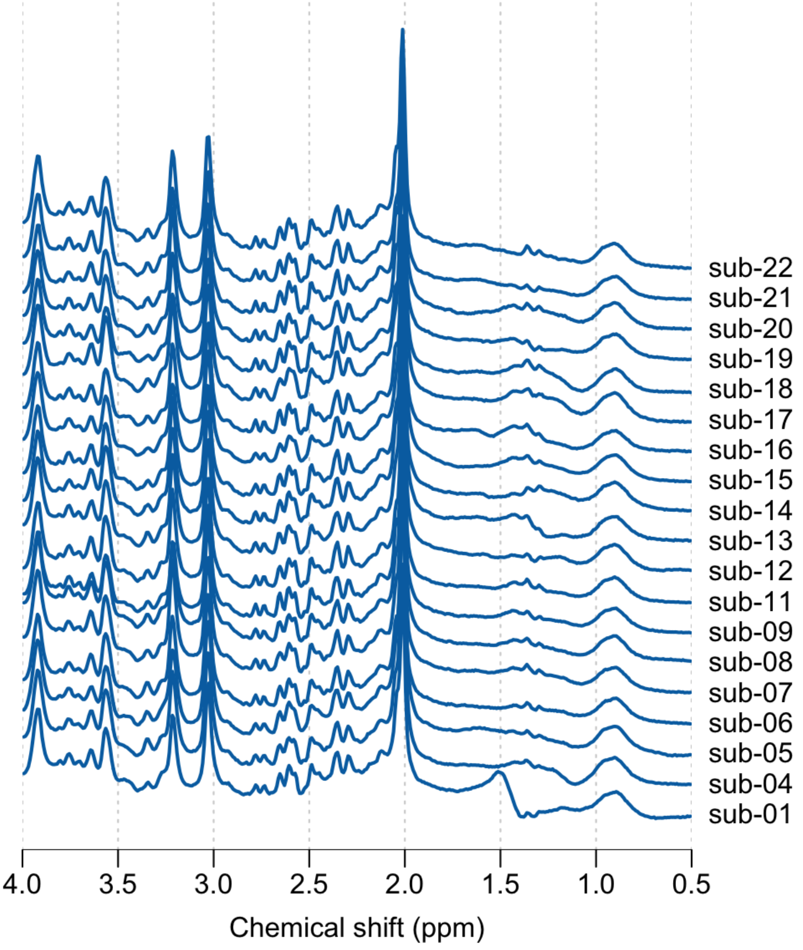

Fig. S5 Mean spectrum for each participant, excluding cases with poor quality spectra.

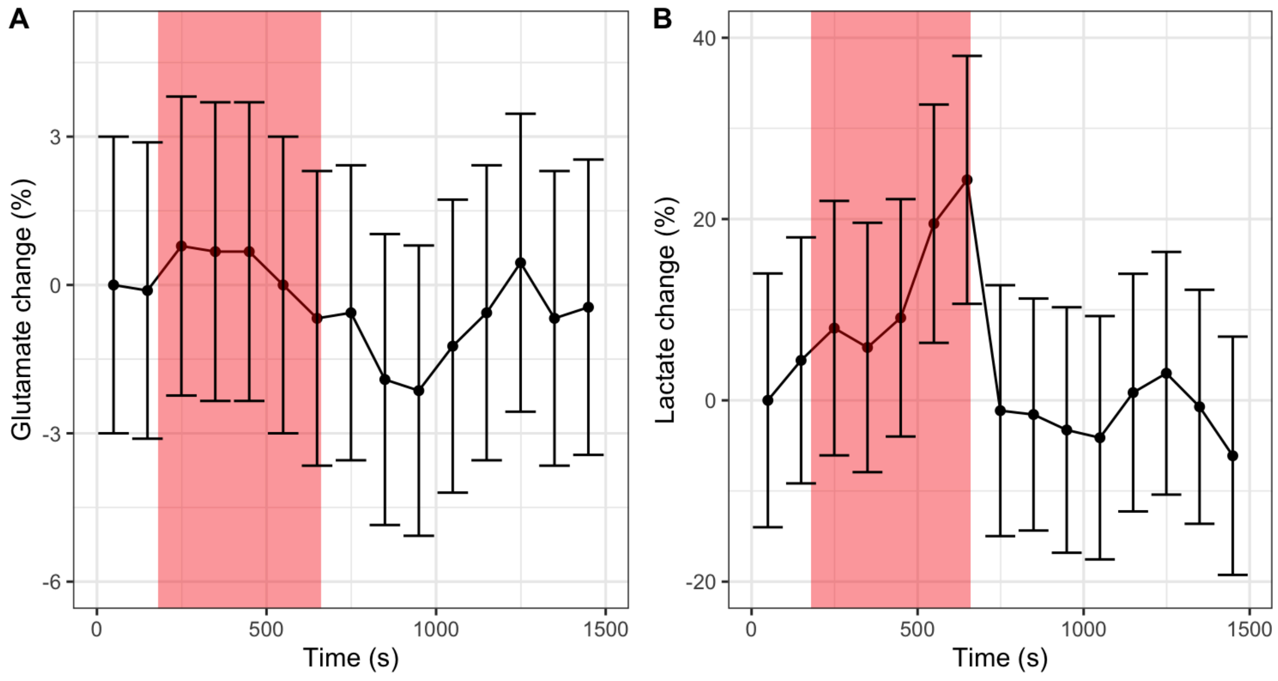

Fig. S6 Time-courses for A) glutamate and B) lactate estimated from spectral fitting of participant averaged spectra using the LCModel method. Error bars represent the standard deviation estimated from Cramer-Rao Lower Bounds. The translucent red region represents the task block.

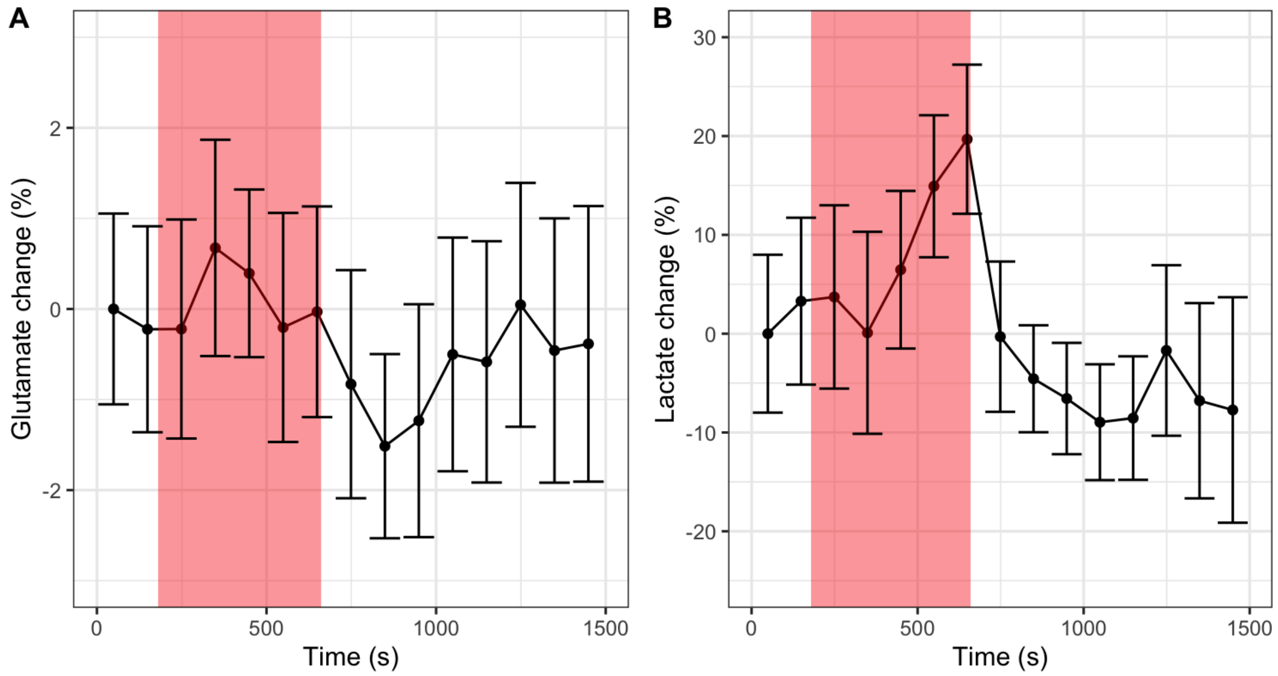

Fig. S7 Mean time-courses for A) glutamate and B) lactate estimated from spectral fitting of individual participant spectra using the LCModel method. Error bars represent the standard error across participants. The translucent red region represents the task block.

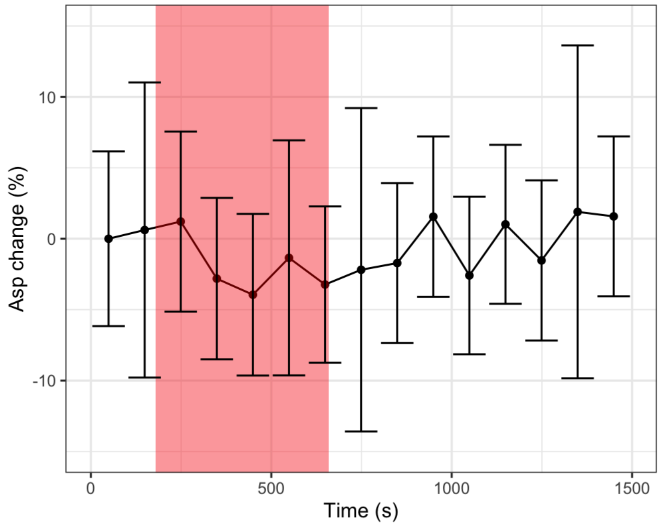

Fig. S8 Time-courses for aspartate estimated from spectral fitting of participant averaged spectra using the ABfit method. Error bars represent the standard deviation estimated from Cramer-Rao Lower Bounds. The translucent red region represents the task block.

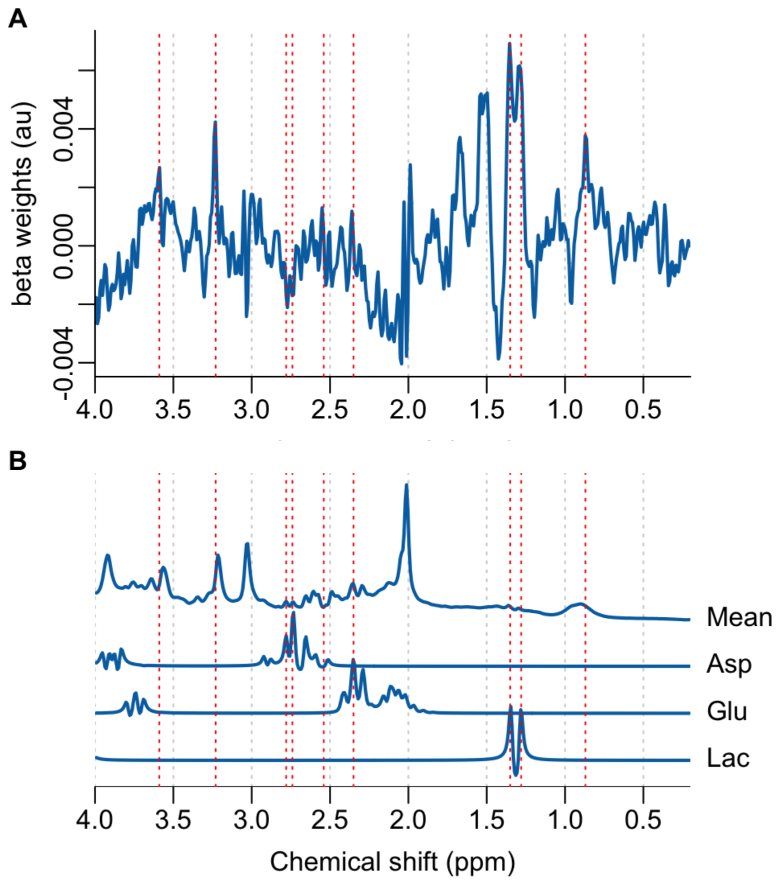

Fig. S9 A) linear model beta weights illustrating spectral regions temporally associated with the functional task, assuming a simple boxcar function delayed by 120 seconds. B) mean fMRS spectrum with simulated glutamate (Glu), lactate (Lac) and aspartate (Asp) signals. Simulated signals are scaled to have similar maximum intensities to aid the assignment of part A). Dashed red lines highlight spectral features at 0.87, 1.28, 1.35, 2.35, 2.54, 2.74, 2.78, 3.23 and 3.59 ppm.

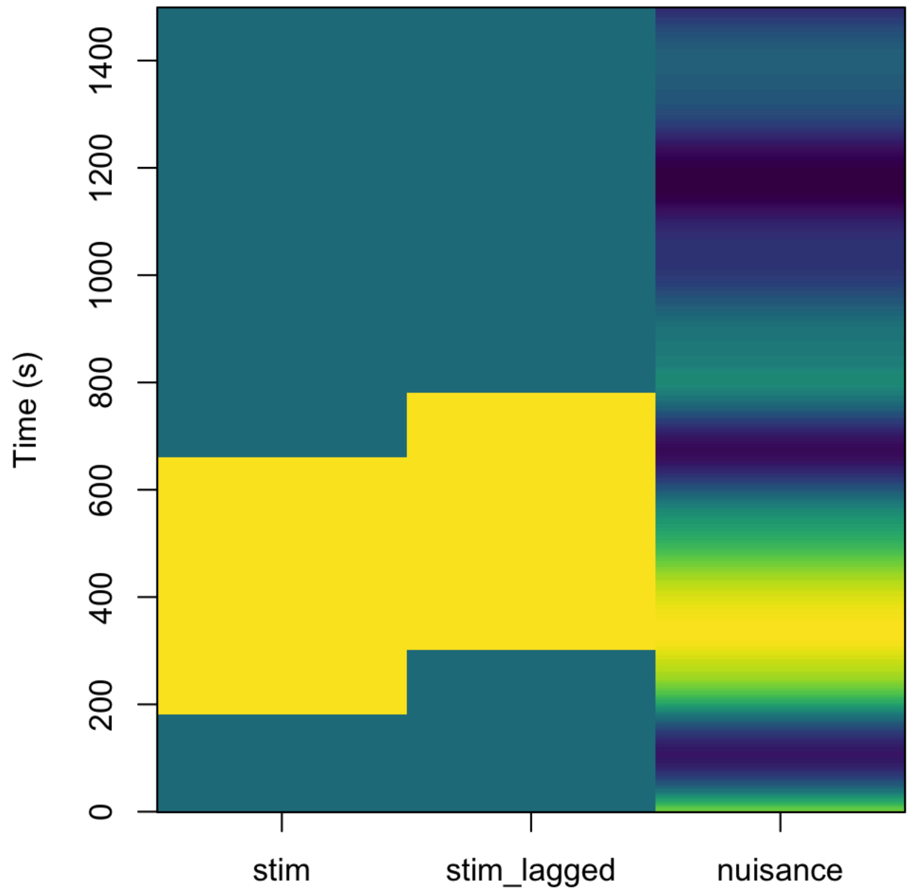

Fig. S10 Regressors used in the GLM analyses.

**1. Hardware**	
a. Field strength [T]	3 T
b. Manufacturer	Siemens
c. Model (software version if available)	PRISMA (VE11C)
d. RF coils: nuclei (transmit/receive), number of channels, type, body part	32 channel receive only head coil
e. Additional hardware	Hand dynamometer
**2. Acquisition**	
a. Pulse sequence	semi-LASER
b. Volume of Interest (VOI)	Hand motor cortex
c. Nominal VOI size	25 x 25 x 25 mm^3
d. TE / TR	28 / 2000 ms
e. Number of Samples Acquired (NSA)	750
f. Additional parameters	BW = 2000 Hz

	Samples = 2048
g. Water Suppression Method	VAPOR
h. Shimming Method	Siemens “brain” shimming
i. Triggering or motion correction method	None
3. Data analysis methods and outputs	
a. Analysis software	spant / ABfit and LCModel
b. Processing steps deviating from quoted reference or product	RATS frequency and zero-order phase correction
c. Output measure<br><br>(e.g. absolute concentration, institutional units, ratio)	Percentage change from baseline
d. Quantification references and assumptions, fitting model assumptions	Default fitting parameters used for spant and ABfit
4. Data Quality	
a. Reported variables<br><br>(SNR, Linewidth (with reference peaks))	SNR & linewidth of tNAA
b. Data exclusion criteria	Visual inspection
c. Quality measures of postprocessing Model fitting (e.g. CRLB, goodness of fit, SD of residual)	CRLB, fit quality number
d. Sample Spectrum	Figure 1 part C and D. Figure S1, S5

Table S1 Minimum Reporting Standards for in vivo Magnetic Resonance Spectroscopy.
